# Supplementary material for: Molecular Ecological Network Complexity Drives Stand Resilience of Soil Bacteria to Mining Disturbances among Typical Damaged Ecosystems in China
Source: Microorganisms. 2020 Mar 19;8(3):433. doi: 10.3390/microorganisms8030433 (PMC7143963; doi:10.3390/microorganisms8030433)
Supplement: Supplementary file 1 [file microorganisms-08-00433-s001.zip › additional file 13í¬Table S1.docx]

Table S1. Mantel test between soil microbial community at the phylum level and environmental factors in the four mining areas

| Envs | Mantel test | | Partial mantel test | |
| --- | --- | --- | --- | --- |
|  | r | p | r | p |
| AAP | 0.3879 | 0.001 | 0.3065 | 0.001 |
| AAT | 0.3430 | 0.001 | 0.2775 | 0.001 |
| pH | -0.0465 | 0.741 | -0.1220 | 0.979 |
| EC | 0.3686 | 0.001 | 0.2899 | 0.001 |
| SOM | -0.0028 | 0.47 | -0.1512 | 0.993 |
| NN | 0.3826 | 0.005 | 0.3793 | 0.003 |
| AN | -0.0025 | 0.418 | -0.2441 | 1 |
| AP | 0.2100 | 0.001 | 0.0599 | 0.123 |
| AK | 0.2942 | 0.001 | 0.1683 | 0.015 |
