# Supplementary material for: Molecular Ecological Network Complexity Drives Stand Resilience of Soil Bacteria to Mining Disturbances among Typical Damaged Ecosystems in China
Source: Microorganisms. 2020 Mar 19;8(3):433. doi: 10.3390/microorganisms8030433 (PMC7143963; doi:10.3390/microorganisms8030433)
Supplement: Supplementary file 1 [file microorganisms-08-00433-s001.zip › additional file 14í¬Table S2.docx]

Table S2. Correlation test between soil microbial community at the phylum level and environmental factors in the four mining areas

|  | AAP | AAT | pH | EC | SOM | NN | AN | AP | AK |
| --- | --- | --- | --- | --- | --- | --- | --- | --- | --- |
| *Acidobacteria* | -0.279(0.037) | -0.208(0.123) | -0.16(0.238) | -0.359(0.00657) | 0.171(0.209) | -0.407(0.00185) | 0.265(0.0485) | 0.341(0.01) | -0.451(0.000484) |
| *Actinobacteria* | -0.549(1.2e-05) | -0.549(1.16e-05) | 0.0871(0.523) | -0.0617(0.651) | 0.115(0.398) | 0.177(0.192) | 0.0426(0.755) | 0.242(0.0719) | 0.027(0.844) |
| *Armatimonadetes* | -0.38(0.00386) | -0.454(0.000446) | 0.558(7.82e-06) | 0.429(0.000971) | -0.283(0.0346) | 0.129(0.343) | -0.485(0.00015) | -0.435(0.000806) | 0.615(4.52e-07) |
| *Bacteroidetes* | 0.548(1.22e-05) | 0.48(0.000179) | 0.0789(0.563) | 0.373(0.00468) | -0.0797(0.559) | 0.256(0.0566) | -0.208(0.124) | -0.448(0.000536) | 0.303(0.0232) |
| *BRC1* | -0.0624(0.648) | 0.00175(0.99) | -0.205(0.13) | -0.392(0.00281) | 0.12(0.377) | -0.131(0.336) | 0.281(0.0359) | 0.462(0.000337) | -0.415(0.00146) |
| *Chlorobi* | 0.384(0.00349) | 0.322(0.0156) | 0.348(0.00854) | 0.612(5.3e-07) | -0.0786(0.565) | 0.115(0.397) | -0.378(0.00409) | -0.557(8.08e-06) | 0.448(0.000534) |
| *Chloroflexi* | -0.204(0.132) | -0.186(0.17) | -0.0577(0.673) | -0.133(0.327) | -0.206(0.128) | -0.321(0.0157) | 0.0138(0.92) | 0.239(0.0763) | -0.0458(0.737) |
| *Cyanobacteria* | 0.141(0.3) | 0.103(0.452) | 0.0284(0.836) | 0.193(0.154) | -0.13(0.341) | -0.0674(0.622) | -0.0567(0.678) | -0.196(0.147) | 0.116(0.395) |
| *Elusimicrobia* | 0.0767(0.574) | 0.0576(0.673) | 0.206(0.128) | -0.00472(0.972) | -0.0436(0.75) | 0.276(0.0398) | -0.0682(0.618) | -0.271(0.0435) | 0.116(0.395) |
| *FBP* | -0.09(0.509) | -0.0321(0.814) | -0.146(0.282) | -0.358(0.00672) | 0.177(0.191) | -0.155(0.253) | 0.317(0.0172) | 0.358(0.00674) | -0.351(0.00802) |
| *Fibrobacteres* | -0.199(0.141) | -0.216(0.109) | 0.348(0.00849) | 0.193(0.155) | -0.121(0.375) | -0.0228(0.868) | -0.209(0.122) | -0.203(0.133) | 0.373(0.0046) |
| *Firmicutes* | -0.129(0.342) | -0.124(0.363) | 0.137(0.316) | -0.115(0.398) | 0.0577(0.673) | 0.047(0.731) | 0.0633(0.643) | -0.0496(0.717) | -0.105(0.441) |
| *GAL15* | 0.401(0.00221) | 0.334(0.0118) | 0.223(0.0988) | 0.412(0.00159) | -0.161(0.237) | 0.0242(0.86) | -0.263(0.0499) | -0.427(0.00102) | 0.271(0.0431) |
| *Gemmatimonadetes* | -0.286(0.0323) | -0.353(0.00768) | 0.433(0.000856) | 0.57(4.54e-06) | -0.256(0.0566) | -0.0252(0.854) | -0.434(0.000821) | -0.369(0.00513) | 0.495(0.000103) |
| *Nitrospirae* | 0.321(0.0159) | 0.288(0.0315) | 0.33(0.0131) | 0.422(0.0012) | -0.0739(0.588) | -0.063(0.645) | -0.228(0.0912) | -0.344(0.0095) | 0.164(0.227) |
| *Planctomycetes* | 0.0205(0.881) | 0.133(0.328) | -0.317(0.0173) | -0.687(4.95e-09) | 0.302(0.0238) | -0.298(0.0258) | 0.538(1.94e-05) | 0.463(0.000325) | -0.667(2e-08) |
| *Proteobacteria* | 0.784(9.05e-13) | 0.77(4.13e-12) | -0.233(0.0842) | 0.137(0.314) | -0.0431(0.752) | 0.106(0.437) | -0.0555(0.685) | -0.242(0.0723) | 0.0823(0.546) |
| *TM6* | -0.0022(0.987) | -0.0145(0.916) | 0.189(0.162) | -0.126(0.355) | 0.071(0.603) | 0.133(0.329) | 0.0265(0.847) | -0.175(0.197) | -0.0467(0.732) |
| *TM7* | -0.226(0.0935) | -0.272(0.0428) | 0.168(0.217) | 0.171(0.208) | -0.166(0.222) | 0.0708(0.604) | -0.25(0.0633) | -0.225(0.0951) | 0.265(0.0488) |
| *Verrucomicrobia* | 0.263(0.0499) | 0.2(0.139) | 0.226(0.0942) | 0.471(0.000253) | -0.291(0.0295) | 0.0833(0.541) | -0.391(0.00287) | -0.602(9.32e-07) | 0.534(2.27e-05) |
| *WS2* | -0.467(0.000289) | -0.519(4.08e-05) | 0.374(0.00456) | 0.352(0.00786) | -0.155(0.255) | 0.053(0.698) | -0.335(0.0117) | -0.153(0.26) | 0.282(0.0355) |
